# Efficient Two-Dimensional Perovskite Solar Cells Realized by Incorporation of Ti_3_C_2_T_*x*_ MXene as Nano-Dopants

**DOI:** 10.1007/s40820-021-00602-w

**Published:** 2021-02-11

**Authors:** Xin Jin, Lin Yang, Xiao-Feng Wang

**Affiliations:** Key Laboratory of Physics and Technology for Advanced Batteries (Ministry of Education), College of Physics, Jilin University, Changchun, 130012 People’s Republic of China

**Keywords:** 2D perovskite solar cells, Ti_3_C_2_T_*x*_ nanosheets, Trap densities, Vertical orientation, Charge transport

## Abstract

Two-dimensional (2D) perovskites solar cells (PSCs) have attracted considerable attention owing to their excellent stability against humidity; however, some imperfectness of 2D perovskites, such as poor crystallinity, disordered orientation, and inferior charge transport still limit the power conversion efficiency (PCE) of 2D PSCs. In this work, 2D Ti_3_C_2_T_*x*_ MXene nanosheets with high electrical conductivity and mobility were employed as a nanosized additive to prepare 2D Ruddlesden–Popper perovskite films. The PCE of solar cells was increased from 13.69 (without additive) to 15.71% after incorporating the Ti_3_C_2_T_*x*_ nanosheets with an optimized concentration. This improved performance is attributed to the enhanced crystallinity, orientation, and passivated trap states in the 3D phase that result in accelerated charge transfer process in vertical direction. More importantly, the unencapsulated cells exhibited excellent stability under ambient conditions with 55 ± 5% relative humidity.

**Supplementary Information:**

The online version contains supplementary material available at 10.1007/s40820-021-00602-w.

## Highlights

- 2D Ti_3_C_2_T_*x*_ MXene nanosheets with high electrical conductivity and mobility were employed as a nanosized additive to prepare 2D perovskite films.
- Doping of Ti_3_C_2_T_*x*_ nanosheets can passivate the defects on the perovskite films surface and accelerate charge transfer process in vertical direction.
- Enhanced crystallinity and orientation of the perovskite films result in a significant increase in short-circuit current density and power conversion efficiency.

**Supplementary Information:**

The online version contains supplementary material available at 10.1007/s40820-021-00602-w.

## Graphical abstract

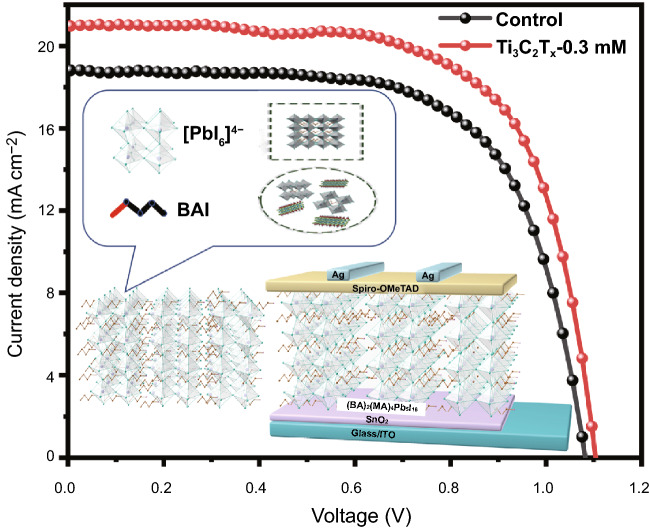

## Introduction

Over the last decade, various efforts have been aimed at converting solar energy to electricity through the photovoltaic effect with novel optoelectrical materials. In particular, organic–inorganic halide perovskites show great potential as excellent light-absorbing materials for photovoltaic devices [1–4]. Thus far, the power conversion efficiencies (PCEs) of organic–inorganic halide perovskite solar cells have been improved from 3.8 to 25.5% through optimization of low-cost solution processing [5, 6]. Although the outstanding photovoltaic performance of perovskite solar cells (PSCs) has attracted worldwide attention, PSCs usually exhibit severe instability to environmental factors, such as moisture, light, and heat, which greatly limits their further commercialization [7]. To address such stability issues, two-dimensional (2D) Ruddlesden–Popper (RP) perovskites with general chemical formula (*A*′)_2_*A*_*n*−1_*M*_*n*_*X*_3*n*+1_, where *n *represents the number of layers of inorganic lead iodide slabs, *A*′, *A*, *M*, and *X* are a bulky long-chain organic spacer, a monovalent cation, a divalent metal cation, and a halide anion, respectively, have proven to be promising [8–11]. 2D RP perovskites are more thermally rigid, with larger cations impeding internal ion movement and allowing sufficient organic groups. That is, these compositions endow the absorber with hydrophobic properties resulting in improved stability in moist environment [12–14]. On the other hand, these structural components are offset by the large exciton binding energy and lower crystallinity [15, 16], which severely affect the separation and transport of carriers and ultimately lead to relatively low current and PCE values.

Current methods for orientation and crystal engineering of 2D RP perovskite films mainly include hot-casting [9], additive-assisted methods, and solvent engineering [17–29]. The hot-casting technique was used to prepare perovskite films, resulting in high-quality films with preferred growth orientation, which facilitates the transfer of charges along the vertical direction of the film; the disadvantage of this approach is that it is difficult to precisely control the temperature. A small amount of Cs^+^ doping resulted in a significant efficiency improvement in 2D RP PSCs [22]. More recently, a PCE of 18.04% with a short-circuit current density (*J*_sc_) of 17.91 mA cm^−2^ was achieved in a (BA)_2_MA_4_Pb_5_I_16_ 2D RP perovskite solar cell based on a water-assisted crystallization process; this translated into an increase in the *J*_sc_ (from 17.61 to 19.01 mA cm^−2^) and PCE (from 13.73 to 15.04%) values of the PSCs [30]. Low current values in 2D PSCs are mainly associated with poor charge transport [18, 31, 32]; therefore, carefully regulating the growth of 2D perovskite films to achieve a better vertical orientation is the key to overcome this problem [23, 33–35]. Despite great advances in the engineering of thin films, the efficiency of 2D RP PSCs is still behind that of their 3D counterparts, especially for the *J*_sc_ [36], and further work is needed to improve their efficiency to meet the commercialization requirement.

MXenes are 2D transition metal carbides and nitrides with a *M*_*n*+1_*X*_*n*_T_*x*_ composition, obtained by etching the *A* (Al, Sn, etc.) layer of the MAX phase, where *M* represents an early transition metal, *X* represents carbon and/or nitrogen, and T_*x*_ indicates surface termination groups (usually –O, –OH, and/or –F) [37]. Ti_3_C_2_T_*x*_, as the first discovered typical MXene, has many excellent properties, including high electrical conductivity, mobility, hydrophilicity, and flexibility [38], which are widely applied in energy storage, supercapacitors, sensors, catalysis, and electromagnetic interference shielding. In addition, Ti_3_C_2_T_*x*_ has been widely applied as a different component in solar cells. For instance, oxidized Ti_3_C_2_T_*x*_ was employed as electrode in dye-sensitized solar cells, resulting in a PCE of 2.66% [39]. Fu et al. [40] reported few-layered Ti_3_C_2_T_*x*_ MXene-contacted Si solar cells with a record PCE of 11.5%. Yu et al. [41] applied Ti_3_C_2_T_*x*_ materials both as electron- and hole-transport layers in organic solar cells and achieved a PCE of 9.06%. We demonstrated the use of UV-ozone-treated Ti_3_C_2_T_*x*_ as electron transport layer (ETL) in PSCs and obtained a highly improved PCE of 17.17% [42]. Moreover, Ti_3_C_2_T_*x*_ was employed as an additive in SnO_2_ [43] or TiO_2_/SnO_2_ [44] multidimensional ETLs, obtaining a high PCE of more than 18%. Guo et al. [45] reported that Ti_3_C_2_T_*x*_ doping in the CH_3_NH_3_PbI_3_ perovskite layer efficiently enhanced the crystal size and charge transfer of the film. Thus, Ti_3_C_2_T_*x*_ MXenes have proven to be promising additives for PCE improvement, highlighting their great potential for application in the field of 2D PSCs, in which Ti_3_C_2_T_*x*_ MXenes remain largely unexplored.

In this study, we for the first time fabricate PSCs employing the 2D (BA)_2_(MA)_4_Pb_5_I_16_ RP perovskite absorber in which Ti_3_C_2_T_*x*_ nanosheets were added as a nano-dopant. Systematic analyses showed that the addition of Ti_3_C_2_T_*x*_ nanosheets in the precursor solution led to a homogeneous perovskite film formation during spin-coating process resulting in spontaneous passivation of grain boundaries. X-ray diffraction (XRD) measurements indicate that the incorporation of an optimal amount of Ti_3_C_2_T_*x*_ nanosheets resulted in an enhanced crystallinity along with a preferential growth perpendicular to the substrate. Meantime, the multiphase coexistence in the 2D perovskite films could be modulated in a preferred order. These characteristics facilitated efficient charge transport, which boost the PCE from 13.69 to 15.71%, as the *J*_sc_ increases from 18.84 to 20.87 mA cm^−2^.

## Experimental Section

### Materials

Lead iodide (PbI2, > 99.99%), methylammonium iodide (MAI, > 99.5%), BAI (99.5%), and lithium-bis (trifluoromethanesulfonyl) imide (Li-TFSI, > 99%) were purchased from Xi’an Polymer Light Technology Corp. NH_4_SCN was purchased from Aladdin. 2,2′,7,7′-tetrakis-(*N*,*N*-di-*p*-methoxyphenylamino)-9,9′-spirobifluorene (Spiro-OMeTAD, > 99.8%), 4-tert-butyl pyridine (tBP, 96%), and ultra-dry anhydrous N,N-dimethylformamide (DMF, 99.8%) and dimethyl sulfoxide (DMSO, 99.7%) were purchased from Sigma-Aldrich. All materials are not further purified before use.

### Preparation of Ti_3_C_2_T_x_ MXene Hydrocolloid

The method is the same as our previous report. The 400-mesh uniform Ti_3_AlC_2_ MAX powder was added into 12 M LiF/9 M HCl solution with continuously stirring for 24 h at room temperature. Specifically, 1.6 g LiF was added to 20 mL 9 M HCl solution at room temperature and stirred for 5 min. Then 1.0 g Ti_3_AlC_2_ MAX powder was slowly added (about 5 min) to the etchant solution and continuously etched for 24 h at room temperature. After etching process, the obtained acid mixture was repeatedly washed over 6 times with deionized water by centrifugation at 8000 rpm for 5 min until the pH of mixture reached about 6. Finally, the slurry was sonicated for 30 min in an ice bath under argon and centrifuged at 3500 rpm for 1 h to separate the multi-layers. The obtained dark supernatant was the colloid solution of Ti_3_C_2_T_*x*_ nanosheets, which could be used directly as the additive in 2D perovskite precursor. To confirm and tune the concentration of Ti_3_C_2_T_*x*_ colloid, a quantitative solution was filtered over a 0.22 µm pore sized cellulose membrane and the concentration of Ti_3_C_2_T_*x*_ was determined to be 11.6 mg mL^−1^ by weighing the peeled-off dried film. Moreover, the solution was diluted to 5.8 mg mL^−1^ by adding deionized water and sonicated for 30 min to control the average size to about 200 nm for better using as additive in perovskite precursor.

### Preparation of 2D Perovskite Precursor Solution

The precursor solution of 2D perovskite BA_2_MA_4_Pb_5_I_16_ (*n* = 5) was prepared by mixing BAI, MAI, PbI_2_, and NH_4_SCN in dimethylsulfoxide (DMSO) and dimethylformamide (DMF) solvents (5:5 volume ratio) with a stoichiometric ratio of 2:4:5:2; the concentration of Pb^2+^ in the precursor solution was 1.2 M. Then, Ti_3_C_2_T_*x*_ hydrocolloid (5.8 mg mL^−1^) was added to the perovskite precursor, at Ti_3_C_2_T_*x*_ concentrations of 0.1, 0.3, 0.5, and 0.7 mM. The solution was stirred for 8 h at 50 °C before use.

### Film Preparation and Device Fabrication

The ITO-coated transparent substrates were cleaned with water, acetone, and alcohol in an ultrasonic bath for 30 min. After drying, the cleaned ITO substrates were treated with UV/ozone for 15 min before use. The SnO_2_ colloid was diluted to 3 wt% by mixing with deionized water and spin-coated onto the ITO substrates at 3000 rpm for 30 s, followed by thermal annealing on a hot plate at 150 °C for 30 min and UV/ozone treatment for 15 min. Then, the UV/ozone-treated substrates were rapidly transferred into a glove box filled with argon, where the 2D perovskite films were fabricated by spin coating the precursor solutions onto the substrates at 4000 rpm for 30 s; during the spin coating process, 300 μL toluene was dripped onto the 2D perovskite film, followed by solvent annealing in DMF atmosphere at 100 °C for 10 min. To prepare the samples containing Ti_3_C_2_T_*x*_, different amounts of Ti_3_C_2_T_*x*_ were introduced in the precursor solution. After the 2D perovskite film was cooled down to room temperature, spiro-MeOTAD was spin-coated on the 2D perovskite film at 4000 rpm for 30 s; the coating solution was prepared by dissolving 72.3 mg spiro-MeOTAD in 1 mL anhydrous chlorobenzene with 28.8 μL tBP and 17.5 μL Li-TFSI (520 mg mL^−1^ acetonitrile) additives. The samples were kept overnight in the dark and dry air at room temperature. Finally, an 80-nm Ag electrode was thermally evaporated on top of spiro-MeOTAD under high vacuum (1 × 10^–4^ Pa). The active device area was determined as 0.04 cm^2^ by overlapping the Ag and ITO electrodes.

### Characterization

#### Thin Film Characterization

UV/Vis absorption spectra were carried out with a Shimadzu UV-1900 spectrophotometer over wavelength range of 300–900 nm. The XRD patterns were recorded on Bruker D8 X-ray diffractometer with Cu Kα radiation (*λ* = 1.5418 Å) at room temperature (25 °C). The data were collected with a 0.02° step size (2*θ*) for 0.2 s. Top-view and cross-sectional scanning electron microscopy (SEM) images were acquired by a field emission scanning electron microscope (Hitachi SU8000) with an energy dispersive spectroscopy (EDS) system. The TEM images were recorded using a JEM-2200FS (JEOL). The roughness of perovskite films was characterized by using AFM (5500, Agilent, Santa Clara, CA). Steady photoluminescence (PL) measurements were carried out by RF-6000 spectrophotometer, while TRPL results were acquired by PL spectrometer (Edinburgh Instruments, FLS 920).

#### Device Characterization

The *J–V* characteristics of perovskite solar cells were carried out by a Keithley 2400 source meter measurement system with an AM 1.5G filter at an illumination intensity of 100 mW cm^−2^, while the solar simulator was calibrated with a Si solar cell and the effective area of the cells was confirmed to be 0.04 cm^2^. The EQE spectra were measured using SOFN 7-SCSpecIII equipped with a 100 W Xe arc lamp, a filter wheel, and a monochromator. The EIS measurements of the devices were carried out on a Princeton electrochemical workstation (Parstat Mc Princeton Instruments Co., Ltd., USA); Z-View Analyst software was used to model the Nyquist plots obtained from the impedance measurements.

## Results and Discussion

### Characterization of Ti_***3***_C_2_T_x_ Nanosheets

**Fig. 1:**
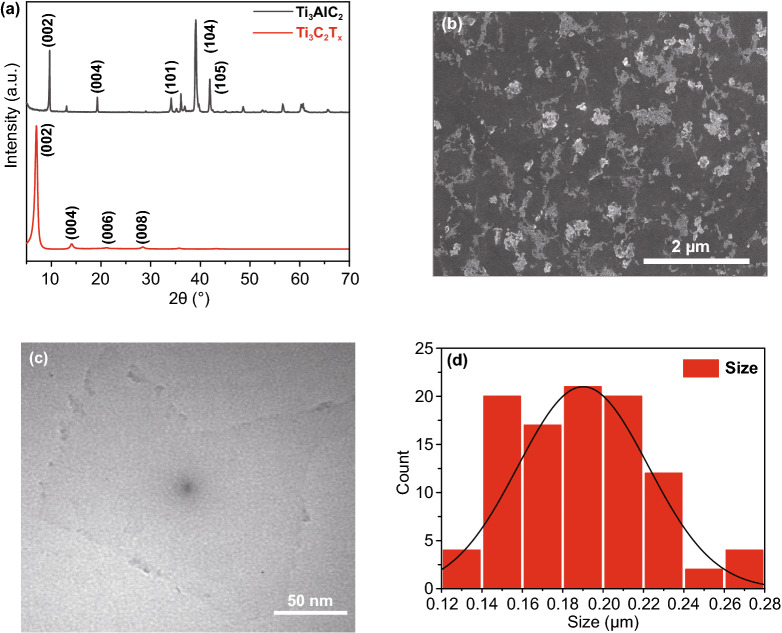
**a** XRD patterns of raw Ti_3_AlC_2_ powder and Ti_3_C_2_T_x_ nanosheets. **b** SEM and **c** TEM images of monolayer Ti_3_C_2_T_*x*_ nanosheets after sonication. **d** Particle size statistics for monolayer Ti_3_C_2_T_*x*_ nanosheets after sonication

Ti_3_C_2_T_*x*_ nanosheets were obtained by etching the original Ti_3_AlC_2_ powder, as described in the experimental section. To confirm the successful transformation of the raw Ti_3_AlC_2_ powder into Ti_3_C_2_T_*x*_ nanosheets, samples of Ti_3_AlC_2_ powder and Ti_3_C_2_T_*x*_ nanosheets were tested by XRD (Fig. 1a). As can be seen in the figure, the disappearance of the strongest diffraction peak of Ti_3_AlC_2_ (104) is accompanied by a shift of the (002) peak from 9.5° to about 7°, which indicates that the Al layer was successfully etched. The diffraction pattern of the Ti_3_C_2_T_*x*_ nanosheets displays four sharp peaks at approximately 7°, 14°, 22°, and 28°, corresponding to the (002), (004), (006), and (008) facets, respectively, which is consistent with previous reports [43]. For the purpose of mixing the Ti_3_C_2_T_*x*_ nanosheets with the perovskite precursor, smaller sized monolayered Ti_3_C_2_T_*x*_ nanosheets were obtained by another 30 min of sonication, as shown in the SEM and transmission electron microscopy (TEM) images in Fig. 1b, c, respectively. The size distribution results for the monolayer Ti_3_C_2_T_*x*_ nanosheets show an average size of approximately 200 nm (Fig. 1d). In order to apply Ti_3_C_2_T_*x*_ nanosheets in perovskite devices, we incorporated Ti_3_C_2_T_*x*_ hydrocolloid into the precursor solution, at concentrations of 0, 0.1, 0.3, 0.5, and 0.7 mM; in the following, the corresponding samples are labeled as control, Ti_3_C_2_T_*x*_-0.1 mM, Ti_3_C_2_T_*x*_-0.3 mM, Ti_3_C_2_T_*x*_-0.5 mM, and Ti_3_C_2_T_*x*_-0.7 mM, respectively.

### Photovoltaic Devices Performance

**Fig. 2:**
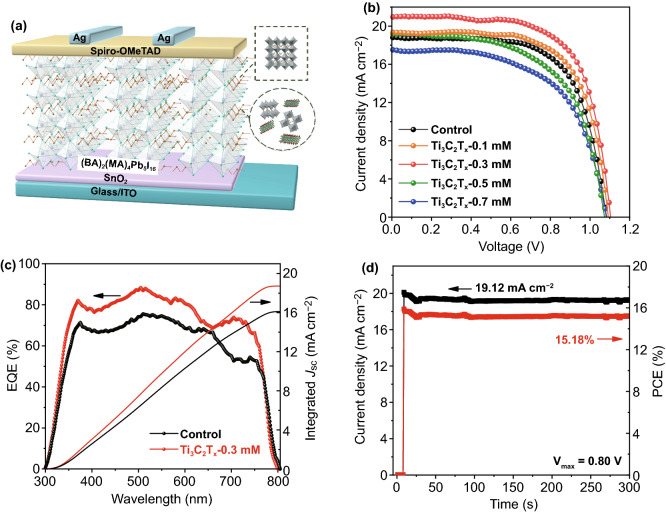
**a** Schematic diagram of devices with the structure of Glass/ITO/SnO_2_/2D perovskite/Spiro-OMeTAD/Ag. **b** *J–V* curves of devices with different amounts of Ti_3_C_2_T_*x*_-doping. **c** EQE spectra and integrated *J*_sc_ of the control and optimized Ti_3_C_2_T_*x*_-doping devices. **d** Stabilized power output and current density at a constant bias of 0.80 V for the Ti_3_C_2_T_*x*_-doping devices

Figure 2a shows an architecture of the present PSCs together with the illustration of introducing Ti_3_C_2_T_*x*_ into the 2D perovskite film. To determine how different Ti_3_C_2_T_*x*_ nanosheet contents affect the photovoltaic performance of 2D (BA)_2_(MA)_4_Pb_5_I_16_ perovskite devices, we fabricated devices with an *n*–*i*–*p* planar architecture on the optically transparent electrode consisting of the indium tin oxide (ITO), the SnO_2_ electron transporting layer (ETL), the 2D perovskite layer, the Spiro-OMeTAD hole transporting layer (HTL), and the Ag back electrodes. The *J–V* curves of devices fabricated with the control, Ti_3_C_2_T_*x*_-0.1 mM, Ti_3_C_2_T_*x*_-0.3 mM, Ti_3_C_2_T_*x*_-0.5 mM, and Ti_3_C_2_T_*x*_-0.7 mM samples are shown in Fig. 2b. Table 1 summarizes the corresponding photovoltaic parameters. Overall, the PCE of the present devices increased and then decreased with increase in the Ti_3_C_2_T_*x*_ content. The increase in the PCE of the devices is mainly attributed to the enhanced current density. In particular, the 2D perovskite device with optimal content of Ti_3_C_2_T_*x*_-0.3 mM showed markedly increased *J*_sc_ (20.87 mA cm^−2^), open-circuit voltage (*V*_oc_) (1.11 V), and fill factor (FF) (67.84%) values, resulting in a greatly improved PCE of 15.71%, compared to a *J*_sc_ of 18.84 mA cm^−2^, *V*_oc_ of 1.09 V, FF of 66.7%, and PCE of 13.69% obtained for the control device. Clearly, the addition of Ti_3_C_2_T_*x*_ nanosheets led to a significant improvement in photovoltaic performance. This improvement in the *J*_sc_ is supported by the external quantum efficiency (EQE) spectrum shown in Fig. 2c. The optimal devices prepared with the Ti_3_C_2_T_*x*_-0.3 mM sample displayed a remarkable change, which can be explained by the marked light absorption associated with Ti_3_C_2_T_*x*_ doping. Based on the EQE spectra, the devices prepared with the control and Ti_3_C_2_T_*x*_-0.3 mM samples had integrated current densities of 19.82 and 17.83 mA cm^−2^, respectively, within an error of 5%. More devices were fabricated to confirm the efficiency repeatability of devices incorporating the Ti_3_C_2_T_*x*_ nanosheets. Figure S1 presents statistics of the relevant photovoltaic parameters obtained from more than 20 devices based on different doping amounts of Ti_3_C_2_T_*x*_ nanosheets, showing that the average PCE of Ti_3_C_2_T_*x*_-0.3 mM was 15.32%, which is much higher than the outstanding reproducibility of the control devices (average PCE = 12.11%). In addition, the photocurrent measured for more than 300 s at a maximum power point (0.80 V) indicates a stable power output, consistent with the *J–V* curves (Fig. 2d).

**Table 1:** Photovoltaic parameters of 2D PSCs with/without Ti_3_C_2_T_*x*_ additive

Samples	*J*_sc_ (mA cm^−2^)	*V*_oc_ (V)	FF (%)	PCE (%)
0 mM Ti_3_C_2_T_*x*_	18.84	1.09	66.70	13.69
0.1 mM Ti_3_C_2_T_*x*_	19.36	1.09	67.39	14.27
0.3 mM Ti_3_C_2_T_*x*_	20.87	1.11	67.84	15.71
0.5 mM Ti_3_C_2_T_*x*_	19.06	1.07	60.28	12.34
0.7 mM Ti_3_C_2_T_*x*_	17.59	1.07	59.39	11.32

### Morphology Characterization

**Fig. 3:**
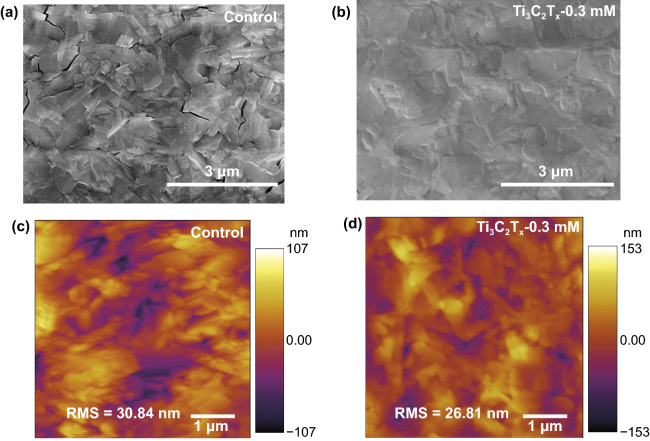
**a**, **b** Top-view SEM and **c**, **d** AFM image of the control and optimized Ti_3_C_2_T_*x*_-doping perovskite films

To understand what happened upon additional Ti_3_C_2_T_*x*_ in perovskite layer, atomic force microscopy (AFM) and SEM were used to inspect the uniformity of the film on the surface of the samples before (control) and after addition of Ti_3_C_2_T_*x*_. In Fig. 3a, b, the top-view SEM images of Ti_3_C_2_T_*x*_-0.3 mM show a homogeneous and dense pattern with almost no holes relative to the control film, which displays a highly rough surface and clear cracks. Probably, the small Ti_3_C_2_T_*x*_ nanosheets are spontaneously distributed at the grain boundaries and defects of the intrinsic perovskite film. Such morphological change upon the Ti_3_C_2_T_*x*_ incorporation must lead to the defect passivation. At other dopant concentrations, observable morphological changes were also observed for Ti_3_C_2_T_*x*_-0.1 mM, Ti_3_C_2_T_*x*_-0.5 mM, and Ti_3_C_2_T_*x*_-0.7 mM (Fig. S3a–c). Increasing the amount of Ti_3_C_2_T_*x*_ in the perovskite layer resulted in a rougher film surface, as further confirmed by AFM (Figs. 3c, d and S3d-f). It is clear that the appropriate amount of Ti_3_C_2_T_*x*_-0.3 mM could induce a smoother film formation. The root-mean-square (RMS) roughness values of the film surface followed the order Ti_3_C_2_T_*x*_-0.7 mM > control > Ti_3_C_2_T_*x*_-0.5 mM > Ti_3_C_2_T_*x*_-0.1 mM > Ti_3_C_2_T_*x*_-0.3 mM, which is consistent with the SEM results. In addition, due to the extremely low content of Ti_3_C_2_T_*x*_ relative to PbI_2_, we could not use energy-dispersive X-ray spectroscopy (EDS) to confirm the presence of Ti_3_C_2_T_*x*_ along the grain boundaries (below the detection limits of EDS). Therefore, we determined the Ti_3_C_2_T_*x*_ location in the film by increasing its amount to 2 mM. The elemental mappings of Pb, I, and Ti are shown in Fig. S4. The figure shows that the Ti species originating from the perovskite precursor were evenly distributed. Based on these observations, it can be concluded that the addition of trace amounts of Ti_3_C_2_T_*x*_ significantly affected the surface morphology of the film. Therefore, the accurate control of the Ti_3_C_2_T_*x*_ content is crucial to improve the photovoltaic performance of the devices.

### Crystalline and Optical Property Analysis

**Fig. 4:**
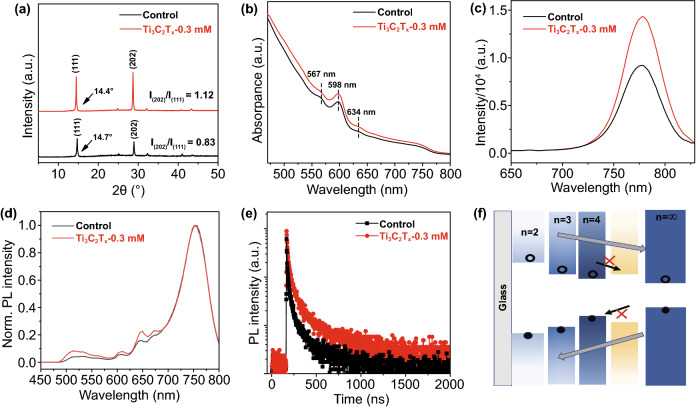
**a** XRD patterns, **b** UV/Vis absorption spectra, **c**, **d** PL spectra excited from front and back side and **e** time-resolved PL spectrums for the control and optimized Ti_3_C_2_T_*x*_-doping perovskite films (on ITO substrates). **f** Illustration of band structure of 2D perovskite film

In general, the excellent photovoltaic performance of 2D perovskite devices is strongly dependent on the crystallization and orientation of the film. Figure 4a displays the XRD patterns of the control and Ti_3_C_2_T_*x*_-0.3 mM perovskite films. The stronger diffraction peaks of Ti_3_C_2_T_*x*_-0.3 mM indicate a higher crystallinity in comparison with the control film. Both films show two primary diffraction peaks at approximately 14° and 28°, corresponding to the (111) and (202) crystal planes. As previously reported in literature [9], the (202) diffraction peaks indicate 2D perovskite films grown perpendicular to the substrate with vertical orientation, while the (111) crystal planes denote an inclined orientation. In the case of control and Ti_3_C_2_T_*x*_-0.3 mM perovskite films, the weaker peak of the (111) crystal plane is accompanied by an enhanced (202) peak, as reflected by (202)/(111) ratios of 0.83 and 1.12, respectively, indicating a better vertical orientation for the Ti_3_C_2_T_*x*_-0.3 mM perovskite film. As demonstrated by Shi et al. [18] using grazing-incidence wide-angle X-ray scattering (GIWAXS), this property promotes the rapid transport of carriers in 2D perovskite films, leading to an increased current. Furthermore, it is worth noting that the overall XRD peaks of the Ti_3_C_2_T_*x*_-based film slightly shifted toward lower diffraction angles, which can be attributed to the expansion of the cells by Ti_3_C_2_T_*x*_ doping. 2D perovskite films have a multiphase nature, and the presence of the characteristic peaks of (111) and (202) planes is likely a consequence of multiphase coexistence [46]. Therefore, the above peak shifts may also be related to the distribution of phases, as discussed below.

2D perovskite films often possess multiple phases due to quantum-well structures [47], as shown by the absorption spectra in Fig. 4b. Multiple absorption peaks appear in the spectra of both control and Ti_3_C_2_T_*x*_-0.3 mM films, such as those at 567, 598, 634, and 775 nm, corresponding to the perovskite phases with *n* = 2, 3, 4, and ∞, respectively. By comparing the intensity of the absorption curves, it can be seen that the Ti_3_C_2_T_*x*_-0.3 mM perovskite films exhibited a stronger absorption at all wavelengths, due to enhanced light scattering and reflection [48]. The direct optical bandgaps of the control and Ti_3_C_2_T_*x*_-0.3 mM perovskite films (Fig. S6) remained almost unchanged.

To further illustrate the multiphase distribution in the 2D perovskite film and the effects of Ti_3_C_2_T_*x*_-0.3 mM doping, PL spectra under front (film) and back (ITO) side excitations are shown in Fig. 4c, d. Upon front excitation, only one individual peak appeared in the spectra of both control and Ti_3_C_2_T_*x*_-0.3 mM films, owing to the region of PL light excitation being confined close to the surface of the perovskite layer. According to previous reports [49], separate peaks originate from large-n phases on the surface of 2D perovskite films. Moreover, the stronger emission intensity of the Ti_3_C_2_T_*x*_-0.3 mM films compared with that of the control reflects the suppression of non-radiative recombination, which can be related to the passivation of defects, in accordance with the SEM findings. Upon back excitation, we could observe phases with different small-n values, which is consistent with previous reports [46]. Surprisingly, Ti_3_C_2_T_*x*_-0.3 mM led to a slight peak enhancement in the small-n phase of the bottom film, which can be explained by the kinetics of film growth after Ti_3_C_2_T_*x*_-doping [18]. Due to the low solubility in the perovskite precursor, Ti_3_C_2_T_*x*_ may largely precipitate at the bottom of the film during spin coating and annealing, acting as a nucleation center for the lower-*n* phases within the bulk, which would increase nucleation of the small-*n* phases and ultimately favor a uniform phase distribution [50]. The enhancement in *J*_sc_ is largely due to this feature. Liu et al. [51] proposed that the *n* = ∞ phase is predominantly distributed at the top of the film, with *n* = 2, 3, 4, and ∞ phases coexisting in the middle, and only small-n phases present at the bottom of 2D perovskite films. However, the small-*n* phases existing in the intermediate mixing region can act as shallow traps in 2D bulk perovskites [51, 52], blocking charge separation and transport, and ultimately greatly affecting the *J*_sc_ of the devices. Ti_3_C_2_T_*x*_ doping can suppress the density of these shallow traps and thus extend the lifetime of the carriers, as shown in Fig. 4e. The time-resolved PL spectrums show that the Ti_3_C_2_T_*x*_-0.3 mM film exhibited longer average lifetimes compared to the control film. The fit formula and relevant parameters of carrier lifetimes are summarized in Table S2. This finding is a good indication that the addition of Ti_3_C_2_T_*x*_ can inhibit non-radiative recombination losses and thus enhance *J*_sc_. The sequential spatial distribution in the order of *n* values is illustrated in Fig. 4f. With an ordered distribution of n values, photogenerated excitons are efficiently dissociated into electrons and holes, shifted in opposite directions, and eventually collected by the electrode.

### Carrier Dynamics

**Fig. 5:**
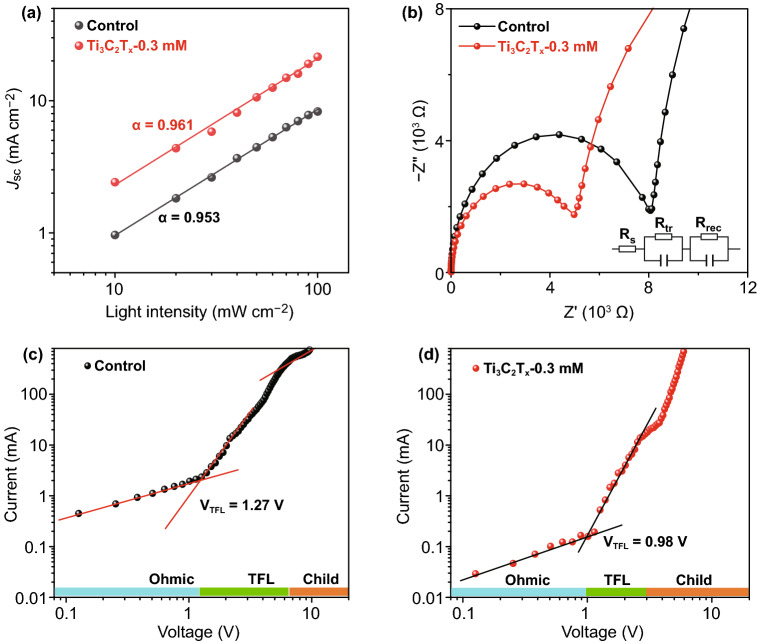
**a** *J*_sc_ versus light intensity and **b** Nyquist plots of EIS with the equivalent circuit for the control and optimized Ti_3_C_2_T_*x*_-doping perovskite devices. **c**, **d** *I–V* curves of the electron-only devices under dark based on the control and optimized Ti_3_C_2_T_*x*_-doping

Current density versus incident light intensity plots were used to illustrate the dynamics of carrier recombination in devices, which follows the relationship$J_{{{\text{sc}}}} \propto I^{\alpha }$, as shown in Fig. 5a. The Ti_3_C_2_T_*x*_-0.3 mM device shows a relationship more closely related to the ideal factor ($\alpha = 1$) on a double-logarithmic scale, with a value of 0.961, compared to a value of 0.953 for the control device; this indicates a lower degree of bimolecular recombination [25], which may be largely attributed to the reduction in trap density, consistent with the SEM observations. In order to quantify the passivation effect of the device upon addition of Ti_3_C_2_T_*x*_ nanosheets, we measured dark *I*–*V* curves of devices with SnO_2_/2D perovskite/PCBM/Ag structure, as shown in Fig. 5c, d. The relevant details of the calculation can be seen in the supporting information. The calculated defect density of the Ti_3_C_2_T_*x*_-incorporating devices was 0.89 × 10^15^ cm^−3^, which is lower than that of the control device (1.16 × 10^15^ cm^−3^), suggesting an improved quality of 2D perovskite film after Ti_3_C_2_T_*x*_-doping.

Subsequently, we examined the characteristics of both devices through electrochemical impedance spectroscopy (EIS) measurements and the Nyquist plots shown in Fig. 5b, with the equivalent circuit model displayed in the inset. Here, *R*_s_ represents the series resistance, which primarily reflects the electrical contacts, wires, and sheet resistance of the electrodes [53]. The charge-transfer properties at the interface are reflected by the charge-transfer resistance (*R*_tr_), whereas *R*_rec_ is related to the recombination of the carriers [54]. It is clear that the *R*_s_ values of the two devices were similar. However, there was a significant variation in the *R*_tr_ value upon Ti_3_C_2_T_x_ doping. Obviously, the Ti_3_C_2_T_*x*_-0.3 mM sample displayed a lower *R*_tr_ value, indicating an optimal charge transfer, which can be explained in terms of the charge-transfer speed at the perovskite/ETL and perovskite/HTL interfaces. The apparent negative relationship between the *R*_tr_ value and Ti_3_C_2_T_x_ doping implies that the charge transport at the surface greatly affects the *J*_sc_ and PCE values.

### Devices Stability

**Fig. 6:**
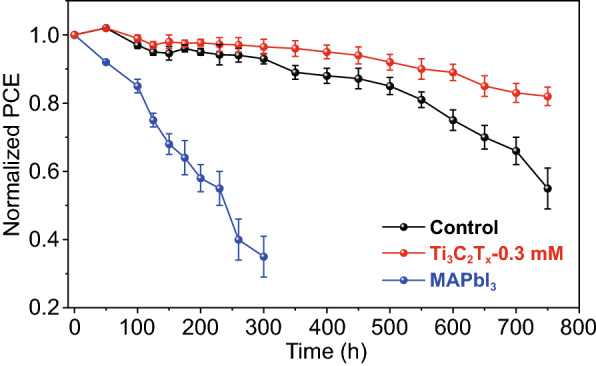
Stability of MAPbI_3_-based, control, and Ti_3_C_2_T_*x*_-doping devices without sealing at air atmosphere with humidity of 55 ± 5%

In contrast to 3D perovskites, excellent stability has always been a characteristic feature of 2D perovskites [12, 55]. The unencapsulated perovskite devices were placed in an environment with 55 ± 5% humidity to monitor their long-term stability. After a certain period of time, the devices were re-exposed to standard light intensity to test their PCE values, as shown in Fig. 6. Overall, the 2D perovskite devices exhibited superior long-term stability in comparison with their 3D counterparts, with almost no degradation after 300 h. Satisfactorily, the Ti_3_C_2_T_*x*_-based devices maintained 80% of their original PCE value after up to 750 h, while the PCE of the control devices was reduced to 40% of the initial value after 700 h of storage. In addition, we examined the thermal stability of 2D perovskite films with/without the presence of Ti_3_C_2_T_*x*_. Figure S7 shows the dependence of absorption intensity reflecting the degradation of 2D perovskite films on continuous heating at 150 ℃ in a N_2_-filled glove box. Compared to Ti_3_C_2_T_*x*_-0 mM perovskite film (original film), Ti_3_C_2_T_*x*_-0.3 mM perovskite film remains almost unchanged after 12 h in the absorption intensity. After 48 h of thermal aging test, the absorption intensity of Ti_3_C_2_T_*x*_-0 mM perovskite film is substantially decreased, whereas no serious decrease in the absorbance was observed in Ti_3_C_2_T_*x*_-0.3 mM perovskite films. This result indicates that the presence of Ti_3_C_2_T_*x*_ improves the thermal stability of 2D perovskite films. It has been reported that when the perovskite devices are exposed to water molecules in the air or continuous high temperature, the additives in Spiro-OMeTAD will diffuse into the perovskite film and the organic components will escape from film to damage the film structure, and eventually leading to film decomposition [56]. The Ti_3_C_2_T_*x*_-containing perovskite precursor solution is spin-coated onto the substrate, and Ti_3_C_2_T_*x*_ in the bulk phase will induce the protonation with CH_3_NH_3_ (MA), because the surface fluorine terminal groups of Ti_3_C_2_T_*x*_ have a strong interaction with hydrogen atoms of MA [45], which results in the superior crystallinity of the Ti_3_C_2_T_*x*_-doped perovskite films (Fig. 4a). In addition, a portion of the Ti_3_C_2_T_*x*_ not inserted into the perovskite lattice will stay at the crystal boundary to passivate defects, as shown in Fig. 3b. Besides, the improved stability of the Ti_3_C_2_T_*x*_-based samples compared to the control sample can also be attributed to the van der Waals interaction that significantly stabilize the framework of 2D perovskite films, resulting in highly oriented crystals with fewer detrimental defects. Moreover, previous studies suggest that species with low-n values in the perovskite layer will produce a passivated layer blocking further degradation [12, 57]. Therefore, the incorporation of Ti_3_C_2_T_*x*_ in perovskite devices provides joint consequence of above effects leading to a higher long-term stability.

## Conclusion

In summary, we have shown that a moderate doping level of Ti_3_C_2_T_*x*_ nanosheets can greatly improve the quality of 2D perovskite (BA)_2_(MA)_4_Pb_5_I_16_ films as well as the photovoltaic performance of the corresponding device, with an increase in PCE from 13.69 to 15.71%, owing to a significant increase in current. The superiority of the Ti_3_C_2_T_*x*_-doped devices is mainly attributed to the intensified crystallinity, vertically oriented growth, and homogeneous phase distribution in the thin film, which ultimately facilitates charge transport. In addition, the Ti_3_C_2_T_*x*_ nanosheets-doped devices exhibit a relatively higher humidity stability than the raw devices, owing to the better crystallinity and passivation effect of perovskite films. This work provides an effective strategy for improving the performance of 2D perovskite films and further expands the applications of Ti_3_C_2_T_*x*_ in photovoltaics.

## Supplementary Information

Below is the link to the electronic supplementary material.

Supplementary file1 (PDF 777 kb)
