# Supplementary material for: Efficient Two-Dimensional Perovskite Solar Cells Realized by Incorporation of Ti3C2Tx MXene as Nano-Dopants
Source: Nanomicro Lett. 2021 Feb 11;13:68. doi: 10.1007/s40820-021-00602-w (PMC8187554; doi:10.1007/s40820-021-00602-w)
Supplement: Supplementary file 1 — Supplementary file1 (PDF 777 kb) [file 40820_2021_602_MOESM1_ESM.pdf]

Supporting Information for

Efficient Two-Dimensional Perovskite Solar Cells Realized by Incorporation of $Ti_3C_2T_x$ MXene as Nano-Dopants

Xin Jin [1,#], Lin Yang [1,#], Xiao-Feng Wang [1,*]

[1]Key Laboratory of Physics and Technology for Advanced Batteries (Ministry of Education), College of Physics, Jilin University, Changchun 130012, PR China

[#]Xin Jin and Lin Yang contribute equally to this work

*Corresponding author. E-mail: xf_wang@jlu.edu.cn (Xiao-Feng Wang)

Supplementary Figures and Tables

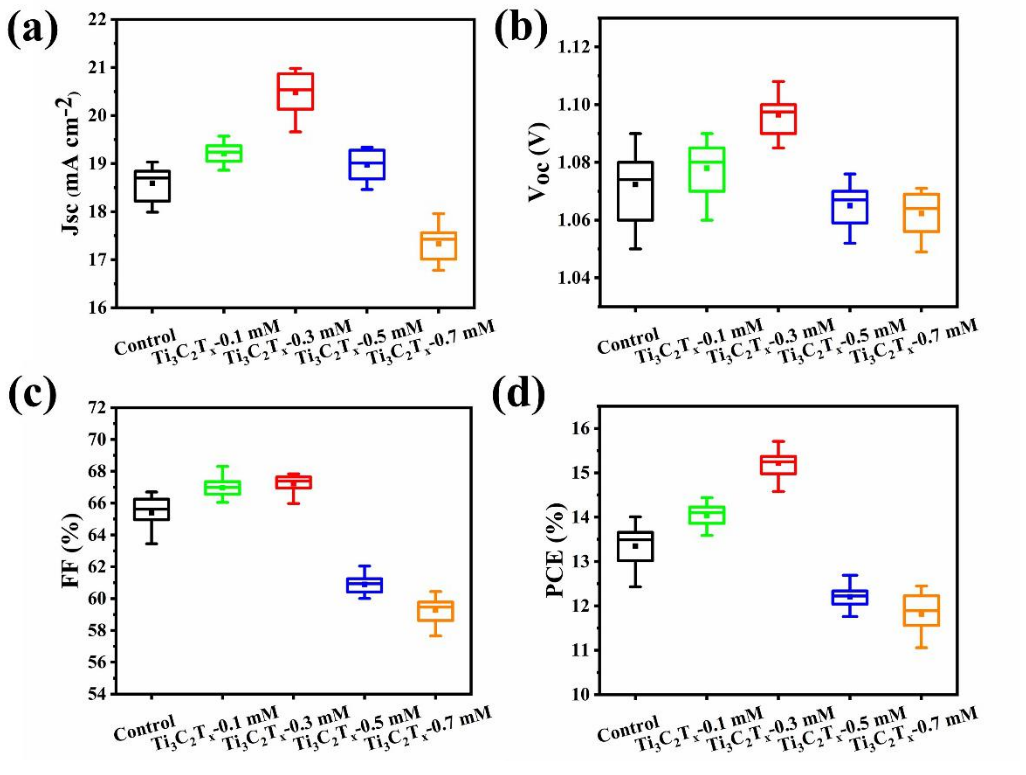

Fig. S1 **a** J_{sc}, **b** V_{oc}, **c** FF and **d** PCE statistic distribution of the control and $Ti_3C_2T_x$-0.1 mM, $Ti_3C_2T_x$-0.3 mM, $Ti_3C_2T_x$-0.5 mM and $Ti_3C_2T_x$-0.7 mM devices

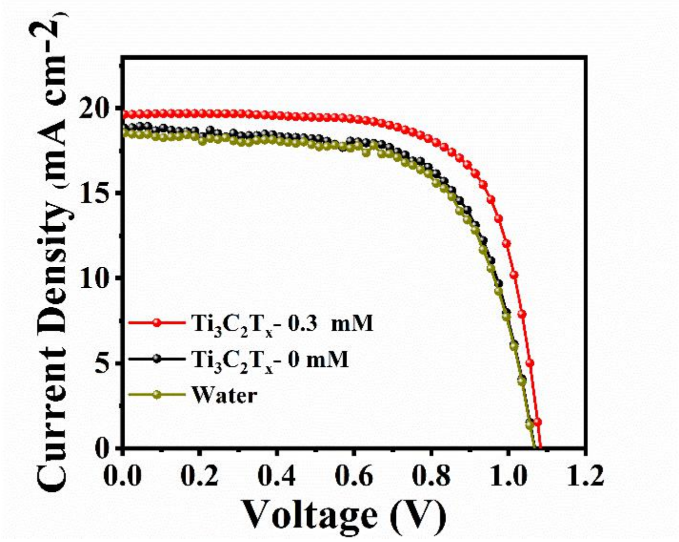

Fig. S2 *J-V* curves of devices with the dopants of water, $Ti_3C_2T_x$-0 mM and $Ti_3C_2T_x$-0.3 mM

Table S1. Performance of PSCs with the dopants of water, $Ti_3C_2T_x$-0 mM and $Ti_3C_2T_x$-0.3 mM

Samples	J_{sc} (mA cm^{-2})	V_{oc} (V)	FF (%)	PCE (%)
$Ti_3C_2T_x$-0 mM	18.53	1.07	64.72	12.83
$Ti_3C_2T_x$-0.3 mM	19.87	1.09	69.11	14.95
Water	18.85	1.07	65.15	13.12

We also carefully analyzed the effect of water on the film. Specifically, the same volume of aqueous solution as MXene hydrocolloid was added to the perovskite precursor solution, and the corresponding device was labeled as water. As a comparison, the original film and the $Ti_3C_2T_x$-doped film labeled as $Ti_3C_2T_x$-0 mM and $Ti_3C_2T_x$-0.3 mM respectively were prepared. The *J-V* curves of devices fabricated with the water, $Ti_3C_2T_x$-0 mM, $Ti_3C_2T_x$-0.3 mM are shown in **Fig. S2**; **Table S1** summarizes the corresponding photovoltaic parameters. Overall, a slight dopant of water in perovskite precursor solution has almost no effect on the perovskite films within the error range, while the addition of $Ti_3C_2T_x$ effectively enhances photovoltaic performance.

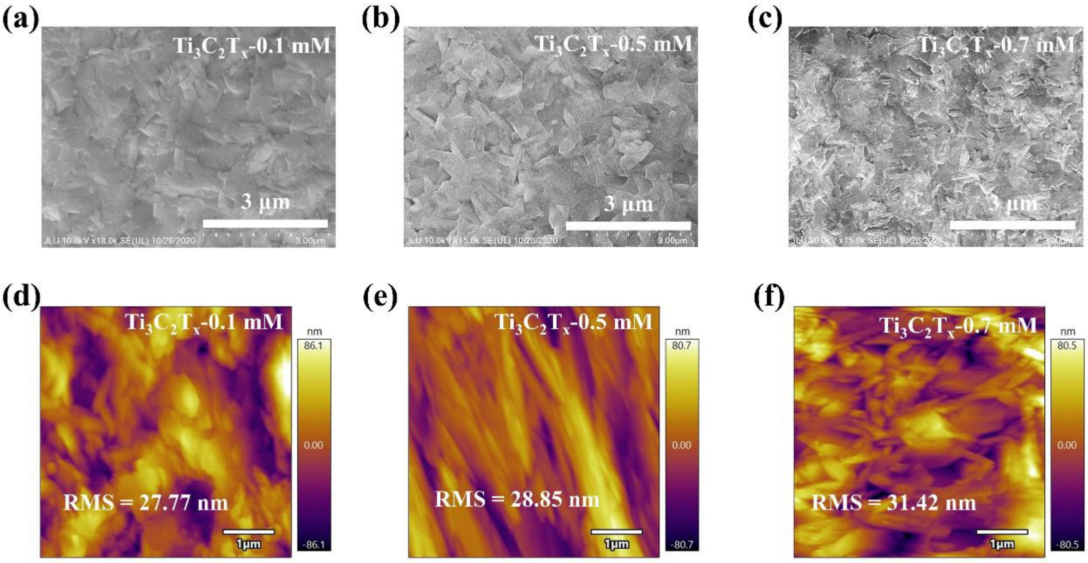

Fig. S3 a, b, c Top-view SEM and **d, e, f** AFM image of the $Ti_3C_2T_x$-0.1 mM, $Ti_3C_2T_x$-0.5 mM and $Ti_3C_2T_x$-0.7 mM perovskite films

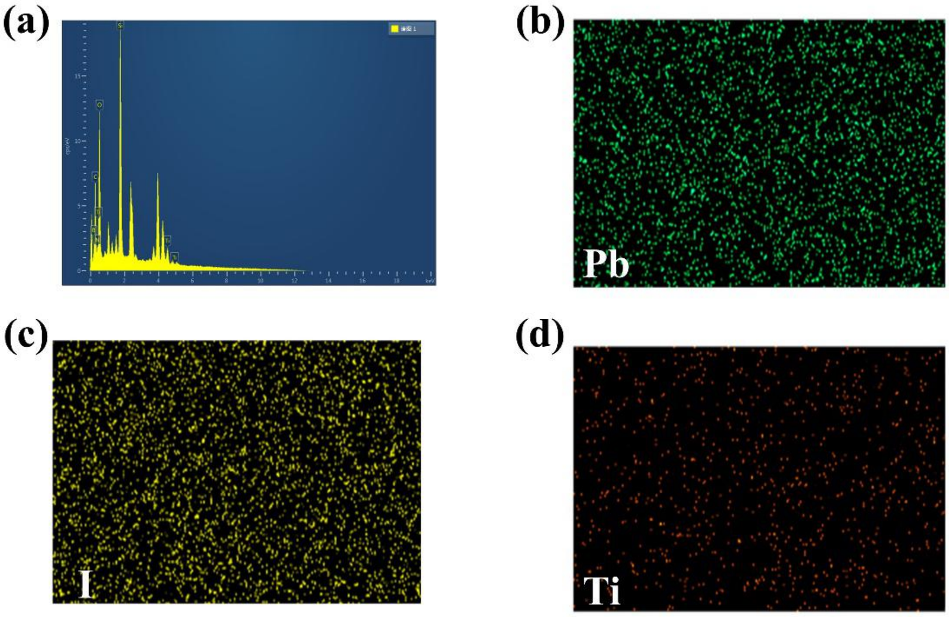

Fig. S4. EDS spectrum of 2D perovskite film with $Ti_3C_2T_x$-2 mM and element mapping of Pb, I and Ti

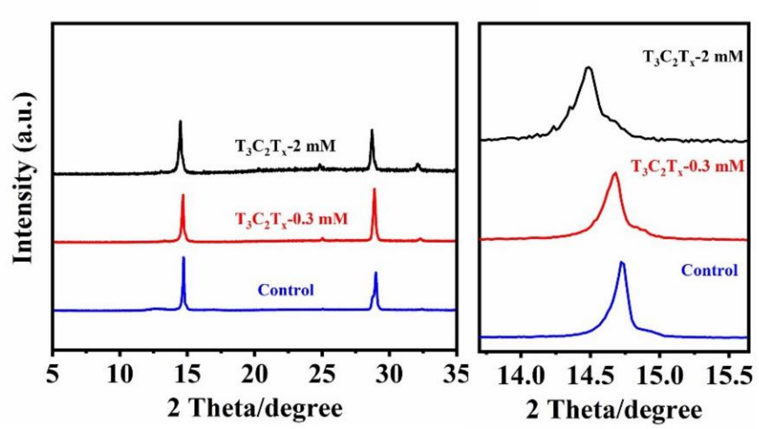

Fig. S5 XRD patterns of the control, $Ti_3C_2T_x$-0.3 mM and $Ti_3C_2T_x$-2 mM perovskite films (on ITO substrates)

In order to prove intuitively the presence of $Ti_3C_2T_x$ in perovskite films, we increased the amount of the additive to 2mM and it can be found that there is a significant shift toward lower angle in the XRD peak.

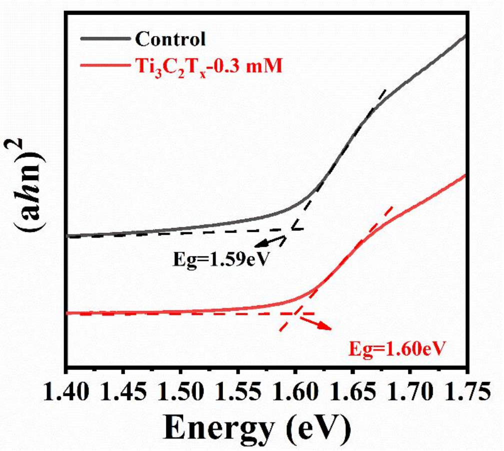

Fig. S6 Tauc plots of control and $Ti_3C_2T_x$-0.3 mM 2D perovskite films

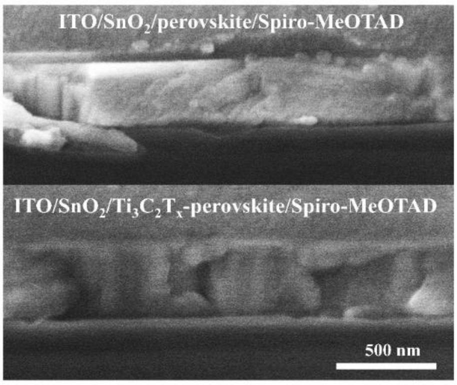

Fig. S7 Cross-sectional SEM image of control and $Ti_3C_2T_x$-0.3 mM-based 2D perovskite devices

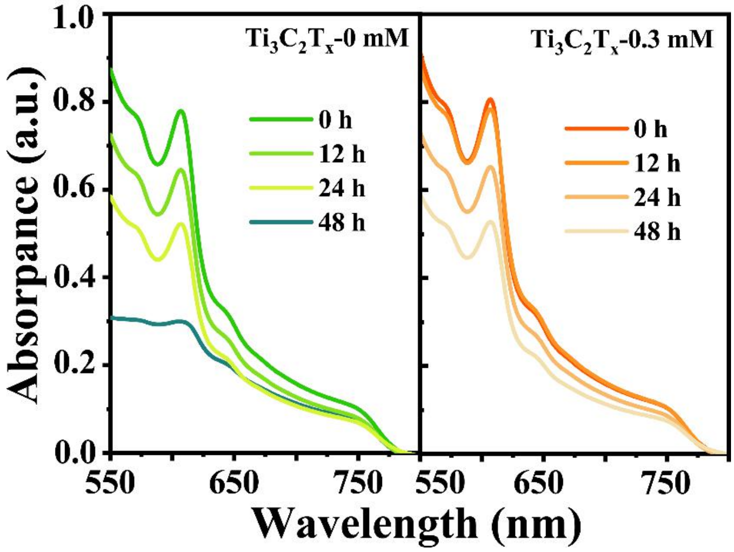

Fig. S8 UV-vis absorption spectra of $Ti_3C_2T_x$-0 mM and $Ti_3C_2T_x$-0.3 mM perovskite films before and after aging at 150°C in a N_2-filled glovebox

Table. S2 Fitted parameters of time-resolved PL spectrums of the control and optimized $Ti_3C_2T_x$-doping perovskite films (on ITO substrate). The average lifetimes (τ_{ave}) are calculated by the equation:

$$\tau_{avg} = \frac{\sum A_i \tau_i^2}{\sum A_i \tau_i}$$

Sample	τ_1 (ns)	τ_2 (ns)	τ_3 (ns)	A_1 (%)	A_2 (%)	A_3 (%)	τ_{ave} (ns)
Control	9.74	48.77	335.8	29.44	41.93	28.63	119.5
$Ti_3C_2T_x$-0.3 mM	8.9	53.4	331.2	24.44	43.94	31.62	130.4

The trap density (N_t) is calculated by using the trap-filled limit voltage (V_{TFL}), following the equation:

$$N_t = \frac{2V_{TFL}\varepsilon_r\varepsilon_0}{qL^2}$$

Where ε_r ($\varepsilon_r = 25$) and ε_0 ($\varepsilon_0 = 8.8 \times 10^{-12}\, Fm^{-1}$) represent the vacuum permittivity and relative dielectric constant, respectively. q is the elementary charge and L is the thickness of the 2D perovskite films.
